# ChatGPT can help guide and empower patients after prostate cancer diagnosis

**DOI:** 10.1038/s41391-024-00864-6

**Published:** 2024-06-26

**Authors:** Harry Collin, Kandice Keogh, Marnique Basto, Stacy Loeb, Matthew J. Roberts

**Affiliations:** 1 Department of Urology, Royal Brisbane & Women’s Hospital, Brisbane, QLD Australia; 2 Faculty of Medicine, The University of Queensland, Brisbane, QLD Australia; 3 Departments of Urology and Population Health, New York University Langone Health, New York, NY USA; 4 University of Queensland Centre for Clinical Research, Brisbane, QLD Australia

**Keywords:** Prostate cancer, Prostate cancer

## Abstract

**Background/Objectives:**

Patients often face uncertainty about what they should know after prostate cancer diagnosis. Web-based information is common but is at risk of being of poor quality or readability.

**Subjects/Methods:**

We used ChatGPT, a freely available Artificial intelligence (AI) platform, to generate enquiries about prostate cancer that a newly diagnosed patient might ask and compared to Google search trends. Then, we evaluated ChatGPT responses to these questions for clinical appropriateness and quality using standardised tools.

**Results:**

ChatGPT generates broad and representative questions, and provides understandable, clinically sound advice.

**Conclusions:**

AI can guide and empower patients after prostate cancer diagnosis through education. However, the limitations of the ChatGPT language-model must not be ignored and require further evaluation and optimisation in the healthcare field.

Patients often self-educate on their health problems, particularly if anxiety impacts information comprehension [1]. Web-based information (e.g. YouTube, TikTok, Instagram) can be of poor quality [2, 3] and poorly actionable (difficult for patients to identify what they can do based on the information presented).

ChatGPT 3.5 is an artificial intelligence (AI), large-language modelling chatbot that generates coherent responses to questions using language patterns from data published prior to September 2021 [4]. ChatGPT responses have been shown to be weakly distinguishable from those of healthcare providers, generating trust in AI chatbots by patients for health information [5]. Response generation does not use direct knowledge so is at risk of misinformation [6]. Thus, this study aimed to evaluate ChatGPT responses and health information quality for prostate cancer patient questions.

ChatGPT 3.5 (https://chat.openai.com/) was asked to generate the 10 most asked questions about prostate cancer. These questions were then compared against the top 25 worldwide Google searches during the 2022 calendar year using Google Trends (https://trends.google.com/trends/; Supplementary Tables 1 and 2). Each question was input into ChatGPT and a response generated (Supplementary Table 3).

A board-certified urologist graded each response as ‘appropriate’ or ‘inappropriate’ advice. The Patient Education Materials Assessment Tool (PEMAT) [7] adjudged content understandability (ability of patients to process and explain key messages) and actionability (ability of patients to identify what they can do based on the information presented) measured as a percentage score of 0–100% (high quality ≥75%). Authorship, currentness and tone were appraised by the QUality Evaluation Scoring Tool (QUEST) [8] scored out of 28. Readability was reported as estimated reading grade level using the Flesch-Kincaid test.

Most (8/10) questions generated by ChatGPT were in the top 25 Google searches related to prostate cancer (Supplementary Table 2). ChatGPT answered these questions with clinically appropriate responses of variable length [mean 206 words, standard deviation (SD) 50 words]. Readability was at a college level.

Seven ChatGPT responses were deemed high quality patient information (Table 1), with a mean PEMAT score of 91.7% for understandability (SD 6.7%). All responses had clear purpose and did not contain distracting information. Most used everyday language with medical terms and numbers presented in an understandable manner, while one response used medical terms without explanation (referring to *BRCA* gene mutations). Six responses employed bullet points, while three responses lacked organisational features. Three responses would have benefited from visual aids. Responses scored a mean PEMAT score of 76% for actionability (SD 12.7%). All responses provided at least one action the user could take. Three responses did not explain the action in explicit steps and only one response addressed the user directly.

**Table 1:** Content quality scores (PEMAT and QUEST) for ChatGPT-generated responses.

Question	PEMAT		QUEST (out of 28)	QUEST (% of total)
	Understandability	Actionability		
1	90%	100%	13	46.4%
2	90%	80%	9	32.1%
3	100%	60%	10	35.7%
4	100%	80%	10	35.7%
5	80%	60%	15	53.6%
6	85%	60%	13	46.4%
7	92%	80%	13	46.4%
8	90%	80%	10	35.7%
9	100%	80%	10	35.7%
10	90%	80%	10	35.7%
Mean	91.7%	76.0%	11.30	40.4%
STD	6.7%	12.6%	2.00	7.2%

QUEST scores for information quality were poor (mean score 11.3 [40.4%], SD 2 [7.2%]), attributable to lack of information authorship, reference to expert sources and indication of currentness. All responses employed cautious vocabulary and eight responses referenced the patient-physician relationship.

Overall, ChatGPT-generated questions matched common patient enquiries and suggests that AI can be a companion for patients who are generally unsure what to ask about prostate cancer.

Responses to these questions were consistently clinically appropriate, signifying that ChatGPT has the potential to offer reliable information to patients. Our study reports higher accuracy (100%) when compared to ChatGPT responses to prostate cancer questions adapted from online patient materials (94.2%) [9], suggesting that patient questions generated by ChatGPT itself can be answered accurately. ChatGPT may be a superior online platform for healthcare advice accuracy compared to others including YouTube [2], TikTok [2] and Instagram [3], known for reporting significant misinformation. However, evaluation with QUEST highlighted issues of ChatGPT information transparency concordant with other studies including a lack of authorship, referencing [10] and currentness [2, 3]. Such transparency limitations were offset by cautious vocabulary to indicate inherent medical uncertainty for patients that are less obvious among online resources.

ChatGPT responses were understandable, reflecting the clear purpose and everyday language in responses. Additionally, each response identified one action the reader could take. While ChatGPT has been shown to confer variable understandability (66.7%) and actionability (40%) across uro-oncology domains [10], our study showed better understandability (91.7%) and actionability (76%), likely due to the relatively basic information need from patients. However, college-level readability may not be applicable for all patients. The absence of explicit steps in some responses may affect the patient’s ability to make informed decisions about their health. Lack of visual aids hindered understandability, which may direct patients to alternative platforms such as TikTok [2] and Instagram [3] where misinformation and poor actionability is more likely to be encountered.

While this study shows that AI is promising for education and patient information, there are clear limitations worth noting. For instance, only 26% of ChatGPT responses to clinical questions on prostate cancer, based on EAU Guidelines, were accurate [11]. ChatGPT-generated questions may not fully capture the breadth or depth for patients from various backgrounds and states of mind. Moreover, we did not account for progressive information gathering, where patients often ask additional questions through dynamic dialogue. Therefore, professional oversight is recommended for clinical applications to ensure clinical safety, effectiveness and reliability. Second, patient preference for education source and format is an important consideration for wider implementation. Patients over 60 years of age have found chatbots usable with low cognitive load [12], but accessibility and effective use is unclear. Additionally, version control of AI chatbots warrant consideration, as ChatGPT 4.0 (from ChatGPT 3.5) is more current and customisable but less accessible due to paywall. Currently, we expect ChatGPT 3.5 to be accessed more by patients, however future research should continually compare various AI models for effective, accurate prostate cancer information. Customisation of AI chatbots for specific tasks presents an opportunity for development of AI models tailored specifically for patient needs.

In conclusion, ChatGPT can offer understandable, clinically appropriate initial advice for newly diagnosed prostate cancer patients. ChatGPT-generated questions reflect what patients typically ask, which can be helpful for patients who otherwise don’t know what to ask. AI chatbots can augment patient education through access to healthcare advice. As AI continues to evolve, mindful integration, ongoing refinement, and collaboration with healthcare professionals and medical databases will be pivotal to harnessing its full potential to empower patients through effective information delivery.

## Supplementary information

Supplementary Table 1

Supplementary Table 2

Supplementary Table 3

## Data Availability

The data analysed in this study is available from the corresponding author upon request.
