# Supplementary material for: ChatGPT can help guide and empower patients after prostate cancer diagnosis
Source: Prostate Cancer Prostatic Dis. 2024 Jun 26;28(2):513–5. doi: 10.1038/s41391-024-00864-6 (PMC12106063; doi:10.1038/s41391-024-00864-6)
Supplement: Supplementary file 1 — Supplementary Table 1 [file 41391_2024_864_MOESM1_ESM.docx]

Supplementary Table 1. The top 25 Google searches related to prostate cancer worldwide in 2022 according to Google Trends.

| **Relative Popularity Rank** | **Related Topics** | **Relative Popularity** |
| --- | --- | --- |
| 1 | Prostate | 100 |
| 2 | Prostate cancer | 97 |
| 3 | Cancer | 97 |
| 4 | Symptom | 13 |
| 5 | Medical Treatment | 10 |
| 6 | Disease cause | 5 |
| 7 | Prostate-specific antigen | 3 |
| 8 | Metastasis | 3 |
| 9 | Radiation therapy | 3 |
| 10 | Signs and symptoms | 2 |
| 11 | Radiation | 2 |
| 12 | Blood | 2 |
| 13 | Benign prostate enlargement | 2 |
| 14 | Surgery | 2 |
| 15 | Urine | 2 |
| 16 | ICD-10 | 2 |
| 17 | Survival rate | 2 |
| 18 | Risk | 2 |
| 19 | Bone | 2 |
| 20 | ICD-10-CM | 2 |
| 21 | Rate | 2 |
| 22 | Colorectal cancer | 2 |
| 23 | Male | 2 |
| 24 | Hormone | 1 |
| 25 | Large intestine | 1 |
