# Supplementary material for: ChatGPT can help guide and empower patients after prostate cancer diagnosis
Source: Prostate Cancer Prostatic Dis. 2024 Jun 26;28(2):513–5. doi: 10.1038/s41391-024-00864-6 (PMC12106063; doi:10.1038/s41391-024-00864-6)
Supplement: Supplementary file 2 — Supplementary Table 2 [file 41391_2024_864_MOESM2_ESM.docx]

Supplementary Table 2. List of questions generated by ChatGPT with corresponding top 25 Google searches related to prostate cancer, obtained with Google Trends. The ranked relative popularity of the 25 Google searches is included.

| **ChatGPT Question** | **Corresponding Google Search**  **(Ranked Relative Popularity)** |
| --- | --- |
| 1. What are the symptoms of prostate cancer? | Symptom (4)  Signs and symptoms (14) |
| 1. What are the causes of prostate cancer? | Disease cause (6) |
| 1. How is prostate cancer diagnosed? | *nil* |
| 1. What are the treatments for prostate cancer? | Medical treatment (5)  Radiation therapy (9)  Radiation (11)  Surgery (14) |
| 1. What is the survival rate for prostate cancer? | Survival rate (17) |
| 1. What is a PSA test, and why is it done? | Prostate-specific antigen (7) |
| 1. What are the risk factors for prostate cancer? | Risk (18) |
| 1. Can prostate cancer be prevented? | *nil* |
| 1. How long does it take for prostate cancer to develop? | Rate (21) |
| 1. What is the difference between early-stage and advanced prostate cancer? | Metastasis (8)  Bone (19) |
| **Non-Represented Google Searches** | |
| Prostate (1)  Prostate cancer (2)  Cancer (3)  Blood (12)  Benign prostate enlargement (13)  Urine (15)  ICD-10 (16)  ICD-10-CM (20)  Colorectal cancer (22)  Male (23)  Hormone (24)  Large intestine (25) | |
