# Supplementary material for: ChatGPT can help guide and empower patients after prostate cancer diagnosis
Source: Prostate Cancer Prostatic Dis. 2024 Jun 26;28(2):513–5. doi: 10.1038/s41391-024-00864-6 (PMC12106063; doi:10.1038/s41391-024-00864-6)
Supplement: Supplementary file 3 — Supplementary Table 3 [file 41391_2024_864_MOESM3_ESM.docx]

Supplementary Table 3. ChatGPT inputs and responses.

| **What are the symptoms of prostate cancer?** |
| --- |
| Prostate cancer may not always cause symptoms in its early stages, and symptoms can vary between individuals. However, some common symptoms of prostate cancer include:   - Difficulty starting or stopping urination - Weak or interrupted urine flow - Frequent urination, particularly at night - Blood in the urine or semen - Pain or discomfort during urination - Pain or discomfort in the pelvic area or lower back - Erectile dysfunction - Decreased force in the stream of urine - Painful ejaculation   It is important to note that these symptoms may also be caused by other conditions, and not all men with prostate cancer will experience these symptoms. If you are experiencing any of these symptoms, it is important to see a healthcare provider for proper evaluation and diagnosis. |
| **What are the causes of prostate cancer?** |
| The exact cause of prostate cancer is not known, but it is believed to be a combination of genetic, environmental, and lifestyle factors. Some of the known risk factors for prostate cancer include:   - Age: Prostate cancer is more common in men over the age of 50, and the risk increases with age. - Family history: Men with a family history of prostate cancer are at a higher risk of developing the disease. - Race and ethnicity: African-American men and men of African descent have a higher risk of prostate cancer than men of other races. - Obesity: Obese men have a higher risk of developing aggressive prostate cancer. - Diet: A diet high in red meat and high-fat dairy products and low in fruits and vegetables may increase the risk of prostate cancer. - Smoking: Smoking has been linked to an increased risk of aggressive prostate cancer. - Exposure to certain chemicals: Exposure to certain chemicals, such as Agent Orange, may increase the risk of prostate cancer.   It's important to note that having one or more risk factors does not mean a man will definitely develop prostate cancer, and some men with prostate cancer may not have any known risk factors. Regular screening and early detection can help with better treatment outcomes for prostate cancer. |
| **How is prostate cancer diagnosed?** |
| Prostate cancer can be diagnosed through several tests and procedures. The diagnostic process typically begins with a physical examination and medical history, followed by one or more of the following tests:   - Prostate-specific antigen (PSA) test: This blood test measures the level of PSA, a protein produced by the prostate gland. Elevated PSA levels may indicate prostate cancer or other conditions, such as prostate enlargement or infection. - Digital rectal exam (DRE): During a DRE, a healthcare provider inserts a gloved finger into the rectum to feel the prostate gland for abnormalities, such as lumps or hard spots. - Biopsy: A biopsy involves the removal of a small sample of prostate tissue for laboratory analysis. A biopsy is typically recommended if the PSA level is elevated or an abnormality is detected during a DRE. - Imaging tests: Imaging tests, such as ultrasound, magnetic resonance imaging (MRI), or computed tomography (CT) scans, may be used to help locate the tumor and determine the stage of the cancer.   Once prostate cancer is diagnosed, additional tests may be needed to determine the extent of the cancer, such as bone scans or positron emission tomography (PET) scans. Treatment options will depend on the stage of the cancer and other individual factors, such as age, overall health, and personal preferences. |
| **What are the treatments for prostate cancer?** |
| The treatment options for prostate cancer depend on several factors, including the stage of the cancer, the patient's age and overall health, and personal preferences. Here are some of the main treatment options for prostate cancer:   - Watchful waiting or active surveillance: For men with low-risk prostate cancer, a healthcare provider may recommend watchful waiting or active surveillance, which involves monitoring the cancer with regular checkups and tests, but not immediately treating it. This approach may be recommended for older men or those with other health conditions that make treatment risky. - Surgery: Surgical removal of the prostate gland, called a radical prostatectomy, may be recommended for men with early-stage prostate cancer. This procedure can be done through an open incision or with minimally invasive techniques, such as laparoscopy or robotic-assisted surgery. - Radiation therapy: Radiation therapy uses high-energy radiation to kill cancer cells. It can be delivered externally, using a machine outside the body, or internally, using radioactive seeds implanted in the prostate gland. Radiation therapy may be used alone or in combination with other treatments. - Hormone therapy: Hormone therapy is used to lower the level of testosterone, a hormone that can fuel the growth of prostate cancer. This can be done with medication or by surgically removing the testicles. - Chemotherapy: Chemotherapy uses drugs to kill cancer cells. It may be recommended for men with advanced prostate cancer that has spread to other parts of the body. - Immunotherapy: Immunotherapy is a type of treatment that helps the immune system recognize and attack cancer cells. It may be used in some cases of advanced prostate cancer. - Other therapies: Other treatments for prostate cancer may include cryotherapy (freezing of the prostate gland), high-intensity focused ultrasound (HIFU), or experimental treatments in clinical trials.   Treatment decisions should be made in consultation with a healthcare provider and should take into account the potential benefits and risks of each option, as well as the patient's individual circumstances and preferences. |
| **What is the survival rate for prostate cancer?** |
| The survival rate for prostate cancer depends on several factors, including the stage of the cancer, the patient's age and overall health, and the treatment options chosen. In general, prostate cancer has a high survival rate, with many men living for years or even decades after diagnosis. According to the American Cancer Society, the relative 5-year survival rate for prostate cancer is nearly 100% for men with local or regional stage cancer (meaning the cancer is confined to the prostate or nearby tissues), and about 30% for men with distant or metastatic stage cancer (meaning the cancer has spread to other parts of the body).  It's important to note that survival rates are estimates based on large groups of people, and individual outcomes may vary. Additionally, survival rates do not take into account newer and potentially more effective treatments that have become available in recent years. Early detection and treatment can improve the chances of survival and reduce the risk of complications from prostate cancer. Regular prostate cancer screening, especially for men with risk factors, is recommended. |
| **What is a PSA test, and why is it done?** |
| The PSA test is a blood test that measures the level of prostate-specific antigen (PSA) in the blood. PSA is a protein produced by the prostate gland, and elevated levels of PSA may indicate prostate cancer or other conditions, such as prostate enlargement or infection.  The PSA test is typically done to screen for prostate cancer or to monitor the progression of the disease in men who have been diagnosed with prostate cancer. It can also be used to detect other prostate problems, such as prostatitis (inflammation of the prostate) or benign prostatic hyperplasia (enlargement of the prostate).  The PSA test is a simple, non-invasive test that involves drawing blood from a vein in the arm. Results are usually available within a few days. While the PSA test can be a useful tool for detecting prostate cancer early, it is not always accurate and can produce false-positive or false-negative results. Other factors, such as age, race, and medications, can also affect PSA levels. Therefore, healthcare providers may use the PSA test in conjunction with other tests, such as a digital rectal exam or biopsy, to diagnose prostate cancer. The decision to undergo PSA testing should be made in consultation with a healthcare provider and should take into account individual risk factors and preferences. |
| **What are the risk factors for prostate cancer?** |
| Several risk factors can increase a man's chances of developing prostate cancer, including:   - Age: Prostate cancer is more common in older men. About 60% of cases occur in men over the age of 65. - Family history: Men with a family history of prostate cancer, particularly a father or brother who had the disease, have an increased risk of developing prostate cancer themselves. - Ethnicity: Prostate cancer is more common in African-American men than in men of other races. It is also more likely to be aggressive and more difficult to treat in this group. - Obesity: Studies have suggested that men who are overweight or obese may have a slightly higher risk of developing prostate cancer, particularly if the cancer is aggressive. - Exposure to certain chemicals: Some studies have suggested that exposure to certain chemicals, such as pesticides, may increase the risk of prostate cancer. - Inflammation of the prostate: Chronic inflammation of the prostate, known as prostatitis, may increase the risk of developing prostate cancer. - Genetics: Inherited gene mutations, such as BRCA1 or BRCA2, have been linked to an increased risk of developing prostate cancer.   While some risk factors for prostate cancer, such as age and family history, cannot be changed, others, such as obesity and exposure to certain chemicals, may be modified through lifestyle changes. It is important for men to discuss their individual risk factors with a healthcare provider and to undergo regular prostate cancer screening, especially if they have risk factors. |
| **Can prostate cancer be prevented?** |
| Currently, there is no sure way to prevent prostate cancer. However, there are some lifestyle changes that may lower the risk of developing prostate cancer or delay its progression:   - Eating a healthy diet: A diet rich in fruits, vegetables, and whole grains, and low in saturated and trans fats, may help lower the risk of prostate cancer. - Maintaining a healthy weight: Being overweight or obese has been linked to an increased risk of prostate cancer, so maintaining a healthy weight through diet and exercise may help lower the risk. - Staying physically active: Regular exercise, such as brisk walking or cycling, may help lower the risk of prostate cancer. - Quitting smoking: Smoking has been linked to an increased risk of several types of cancer, including prostate cancer, so quitting smoking may help lower the risk. - Getting regular prostate cancer screening: Early detection and treatment of prostate cancer can improve outcomes and reduce the risk of complications.   While these lifestyle changes may help lower the risk of developing prostate cancer, they cannot completely eliminate the risk. It is important for men to discuss their individual risk factors with a healthcare provider and to undergo regular prostate cancer screening, especially if they have risk factors. |
| **How long does it take for prostate cancer to develop?** |
| The development of prostate cancer is generally slow, and it can take several years for the cancer to grow large enough to cause symptoms or be detected by screening tests. In some cases, prostate cancer can grow very slowly and may never cause any symptoms or health problems.  The time it takes for prostate cancer to develop can vary depending on factors such as the individual's age, overall health, and the aggressiveness of the cancer. In some cases, prostate cancer can grow rapidly and spread to other parts of the body, while in other cases, it may grow very slowly and remain localized to the prostate gland.  The most common method for detecting prostate cancer is through routine screening, which involves a PSA blood test and a digital rectal exam. Men with an elevated PSA level or abnormal findings on a digital rectal exam may be referred for further testing, such as a prostate biopsy.  Regular prostate cancer screening is recommended for men beginning at age 50, or earlier for men with certain risk factors, such as a family history of prostate cancer or African-American race. It is important for men to discuss their individual risk factors and screening schedule with a healthcare provider. |
| **What is the difference between early-stage and advanced prostate cancer?** |
| Early-stage prostate cancer refers to cancer that is confined to the prostate gland and has not spread to other parts of the body. Early-stage prostate cancer is typically detected through routine screening tests such as the PSA blood test or a digital rectal exam, and may not cause any symptoms.  Advanced prostate cancer, on the other hand, refers to cancer that has spread beyond the prostate gland to other parts of the body, such as the bones, lymph nodes, or other organs. Advanced prostate cancer may cause symptoms such as bone pain, weight loss, fatigue, and difficulty urinating.  Prostate cancer is typically staged based on the extent of the cancer, ranging from stage I, which is confined to the prostate gland, to stage IV, which has spread to other parts of the body. Early-stage prostate cancer is typically treated with surgery, radiation therapy, or active surveillance, while advanced prostate cancer may be treated with hormone therapy, chemotherapy, immunotherapy, or other treatments.  It is important for men to undergo regular prostate cancer screening to detect the cancer early, when it is more likely to be treatable and may have a better prognosis. Men who have symptoms of prostate cancer, such as difficulty urinating, blood in the urine or semen, or bone pain, should see a healthcare provider for evaluation. |
